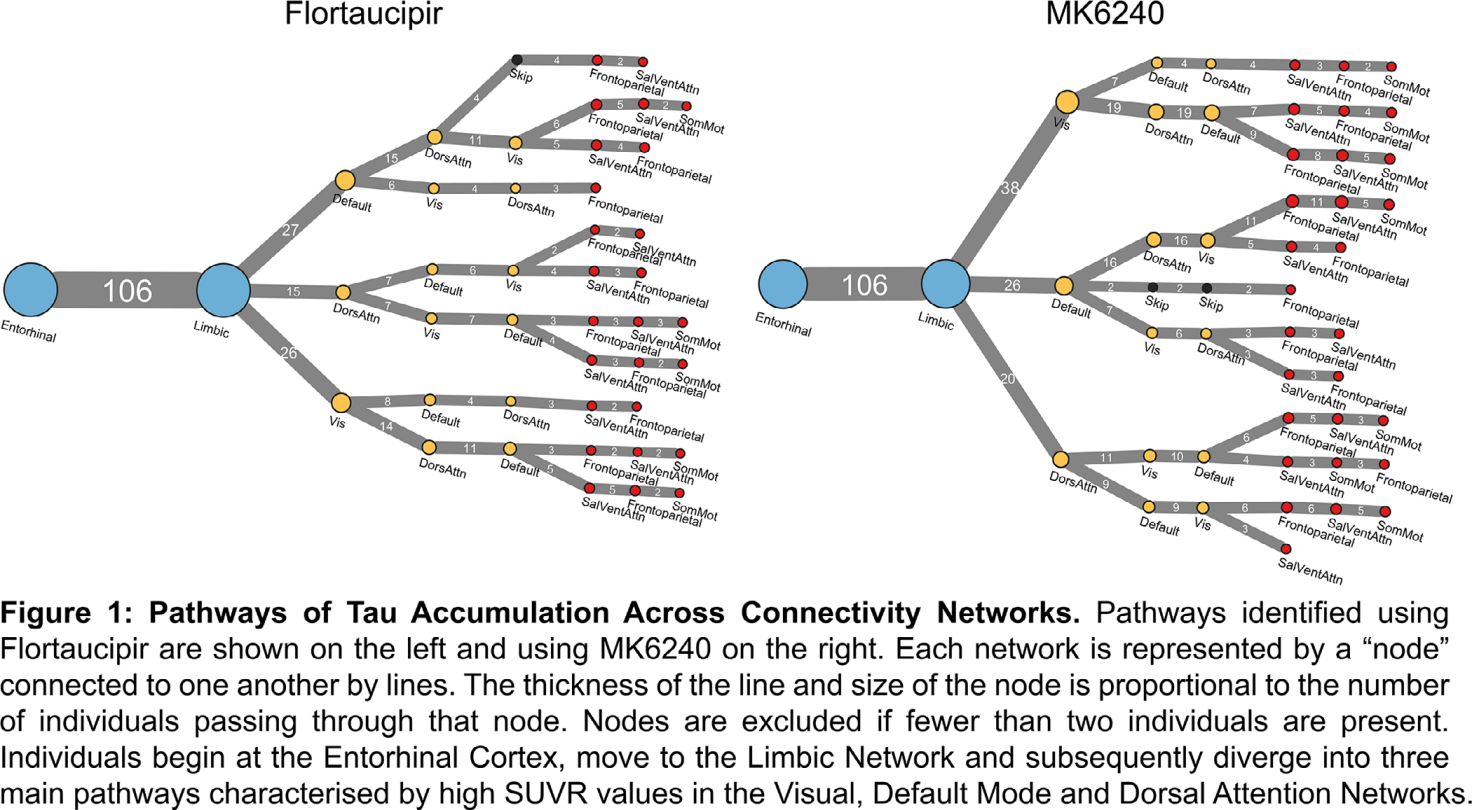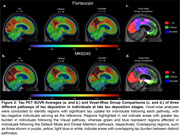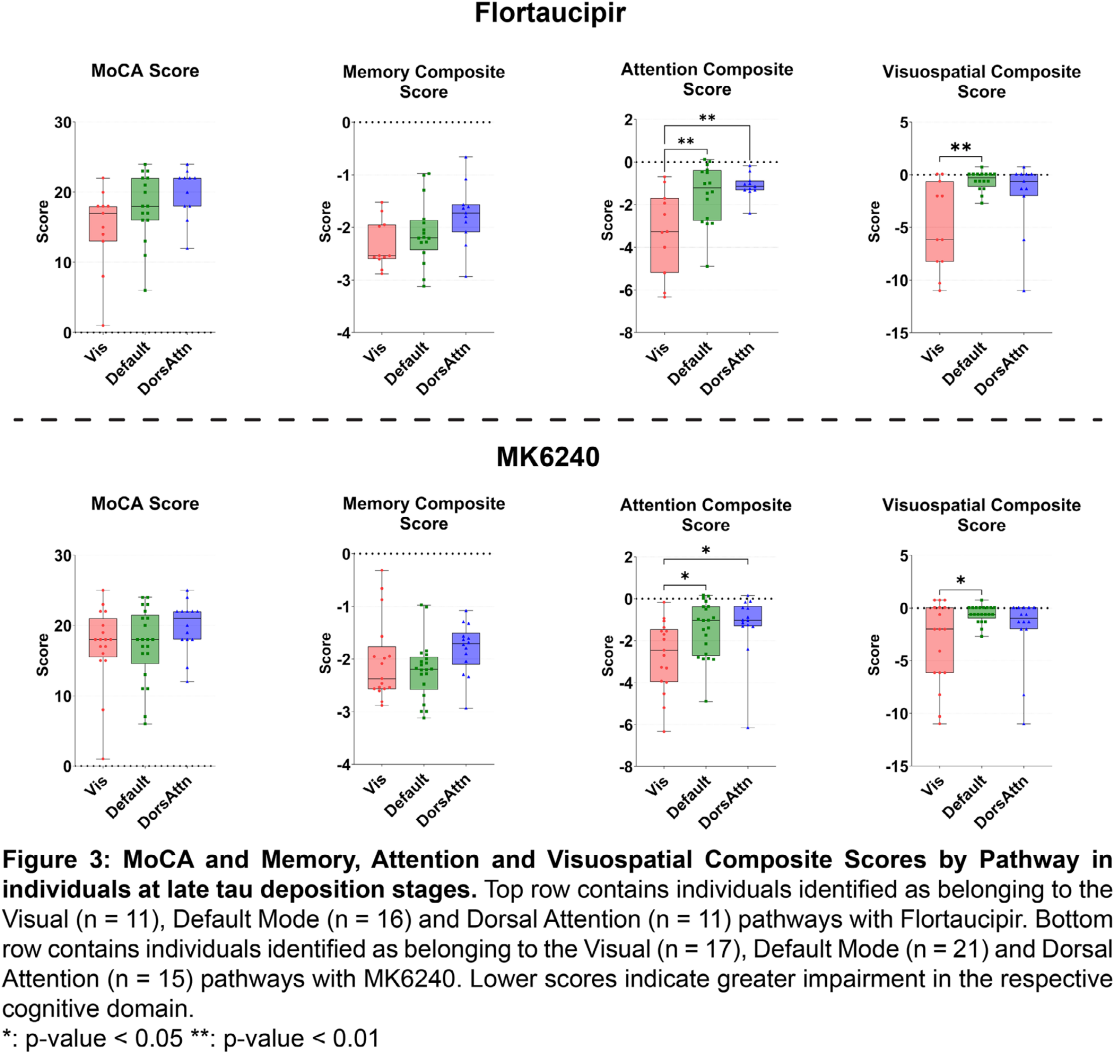# Different Patterns of Propagation of Tau Tangle Pathology in Typical Alzheimer's Disease Determine Clinical Sub‐Phenotypes

**DOI:** 10.1002/alz70862_110836

**Published:** 2025-12-23

**Authors:** Rayan Mroué, Guilherme Povala, Bruna Bellaver, Pamela C.L. Ferreira, Guilherme Bauer‐Negrini, Firoza Z Lussier, Livia Amaral, Marina Scop Madeiros, Emma Ruppert, Andreia Silva da Rocha, Matheus Scarpatto Rodrigues, Markley Silva Oliveira, Carolina Soares, Joseph C. Masdeu, David N. soleimani‐Meigooni, Juan Fortea, Val J Lowe, Hwamee Oh, Belen Pascual, Brian A. Gordon, Pedro Rosa‐Neto, Suzanne L. Baker, Tharick A Pascoal

**Affiliations:** ^1^ University of Pittsburgh, Pittsburgh, PA USA; ^2^ Houston Methodist Research Institute, Houston, TX USA; ^3^ Memory and Aging Center, Weill Institute for Neurosciences, University of California San Francisco, San Francisco, CA USA; ^4^ Sant Pau Memory Unit, Hospital de la Santa Creu i Sant Pau, Institut de Recerca Sant Pau ‐ Universitat Autònoma de Barcelona, Barcelona Spain; ^5^ Mayo Clinic, Rochester, MN USA; ^6^ Brown University, Providence, RI USA; ^7^ Washington University in St. Louis, School of Medicine, St. Louis, MO USA; ^8^ McGill University Research Centre for Studies in Aging, Douglas Research Centre, Montreal, QC Canada; ^9^ Lawrence Berkeley National Laboratory, Berkeley, CA USA

## Abstract

**Background:**

One of the hallmarks of Alzheimer’s disease (AD) is the progressive spread of tau pathology. However, heterogeneity in the spatial patterns of tau deposition at the individual level may contribute to distinct clinical presentations. Here, we investigated the different patterns of tau deposition across disease progression and the different cognitive profiles linked to these patterns.

**Method:**

We investigated 106 tau PET positive individuals (16% cognitively unimpaired, 84% amnestic cognitively impaired) from the HEAD study using head‐to‐head Flortaucipir and MK6240 tau PET. For each tracer, we extracted the SUVR values from seven major brain networks: Limbic, Visual (Vis), Default Mode (Default), Dorsal Attention (DorsAttn), Frontoparietal, Somatomotor (SomMot) and Salience Ventral Attention (SalVentAttn) as well as the Entorhinal cortex. Pathways were identified by ranking regions from highest to lowest SUVR, with the region displaying the highest SUVR value being defined as the peak region. Individuals in late stages of tau deposition displaying distinct pathways were compared. Image averages and voxel‐wise group comparisons using tau negative individuals as the reference were generated for each pathway and tau PET tracer. Cognitive differences were assessed using ANOVA for MoCA score and domain‐specific composite scores for Memory, Attention and Visuospatial skills.

**Result:**

In all individuals and tracers, tau in the Entorhinal cortex progressed to the Limbic network, supporting a common starting point for tau propagation. Beyond these initial stages, tau propagation followed three main distinct pathways following either the Vis, Default or DorsAttn networks (Figure 1). Voxel‐wise comparison between individuals across these three pathways captured clear and similar differences in tau PET uptake as measured with Flortaucipir and MK6240. (Figure 2). While MoCA and Memory scores did not differ between groups, individuals following the Vis network exhibited significantly lower Attention scores compared to those in the Default and DorsAttn networks. Additionally, individuals following the Vis network exhibited lower Visuospatial scores compared to the Default network group (Figure 3).

**Conclusion:**

Our results indicate a model in which, although tau progresses hierarchically, its deposition peaks mainly follow specific networks, which drive tau deposition and, consequently, the clinical manifestations of AD.